# Detection rates and diel activity patterns of four understudied felids from Borneo

**DOI:** 10.1002/ece3.70301

**Published:** 2024-09-15

**Authors:** Maximilian L. Allen, Andrew T. L. Allan

**Affiliations:** ^1^ Prairie Research Institute, Illinois Natural History Survey University of Illinois Champaign Illinois USA; ^2^ Department of Anthropology Durham University Durham UK

**Keywords:** Borneo, *Catopuma badia*, detection rates, diel activity, *Neofelis diardi*, *Pardofelis marmorata*, *Prionailurus javenensis*, *Prionailurus planiceps*

## Abstract

Carnivore guilds are charismatic and have vital and irreplaceable roles in their native ecosystems, yet many of these species are threatened and remain understudied. Borneo is a biodiversity hotspot that hosts a rich diversity of endemic wildlife but is threatened by deforestation and habitat loss. Using cameras placed by the Smithsonian Institution in Sabah, Borneo, we assessed the detection rates and diel activity patterns of the native felid species. Across 51 camera trap sites between 2016 and 2019, felids were detected 55 times across a combined 9958 trap nights, including 20 independent detection events for Sunda leopard cats, 15 for Sunda clouded leopards, 12 for Borneo bay cats, and 5 for marbled cats, with no detections of flat‐headed cats. Collectively, this demonstrates the challenge of sampling cryptic species that have declined due to habitat loss and conflict with humans. Despite this, we show that Borneo bay cats and marbled cats exhibited different diel activity patterns than Sunda cloud leopards, suggesting the smaller species use temporal displacement to avoid competition and predation. Sunda leopard cats exhibited broadly similar activity patterns to Sunda clouded leopard, potentially because the two species typically occupy different dietary and habitat niches. These results demonstrate the importance of devoting future research towards monitoring these species and understanding the mechanisms by which they co‐exist.

## Graphical abstract

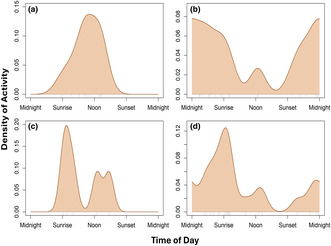

## 1 INTRODUCTION

Carnivores play a critical role in maintaining the health and stability of ecosystems (Estes et al., 2011) and are often among the species most affected by anthropogenic activities and development (Weiss et al., 2023). Felids are particularly charismatic, with strong effects on ecosystems, yet paradoxically many felid species remain understudied. Demographic traits and their variation, including diel activity patterns, represent key aspects of how mammals respond and adapt to anthropogenic changes (Gaynor et al., 2018; Weiss et al., 2023). Moreover, diel activity patterns are fundamental to predator–prey interactions (Allen et al., 2021), influencing the dynamics of these relationships within ecosystems (Gervasi et al. 2013). As such, comprehensive studies of carnivore behavior and ecology are imperative for developing informed conservation strategies, especially in biodiversity hotspots that are threatened by anthropogenic activities.

The island of Borneo is a biodiversity hotspot that hosts a rich diversity of endemic wildlife (Woodruff, 2010), including 230 mammal species (de Bruyn et al., 2014). Unfortunately, however, the island has become a global hotspot for deforestation and habitat loss. Despite formerly containing one of Earth's largest undisturbed tropical forests–an estimated 18.7 Mha of old‐growth forest was cleared between 1973 and 2015 (Gaveau et al., 2016), with additional logging of secondary forests. The principal driver of this change was conversion to industrial plantations, particularly pulpwood and oil palm (*Elaeis guineensis*) (Cushman et al., 2017; Gaveau et al., 2016). These losses are exemplified in the Malaysian state of Sabah with 39.5% of the total forest area being converted to non‐forest between 1973 and 2010 (Gaveau et al., 2014). As a result of these intense anthropogenic impacts, the natural habitats available for wildlife have been reduced dramatically (Ocampo‐Peñuela et al., 2020). Oil palm forests are particularly problematic for biodiversity, being known to support fewer species than other tree crops (Fitzherbert et al., 2008). Road development increases access to remote locations, contributing to the depletion of mammal populations via hunting (Brodie et al., 2015). Although hunters rarely target felids, incidental hunting has been reported (Mohd‐Azlan et al., 2017) and larger predators are affected by prey depletion (Wolf & Ripple, 2016). Given that the decline of predators can lead to trophic downgrading (Estes et al., 2011), further losses to Borneo's fragile predator populations could dramatically alter ecological communities on the island (Ratnayeke et al., 2018).

Asian rainforests are characterized by a higher number of sympatric mammalian carnivores than observed in other tropical regions (Corlett 2007). There are a total of 80 species of carnivores in the Asian region, including five wild felid species in Borneo alone. The five wild felids known to inhabit Borneo are among the least‐studied felids on Earth and each species is of conservation concern, necessitating further study. Most previous studies concur that Borneo bay cats (*Catopuma badia*; 3–4 kg) are diurnal in their diel activity (Hearn et al., 2018; Mohd‐Azlan et al., 2023; Sastramidjaja et al., 2015). Flat‐headed cats (*Prionailurus planiceps*; 2 kg) are considered cathermal in their activity patterns (Mohd‐Azlan et al., 2023). Most studies suggest that marbled cats (*Pardofelis marmorata*; 2–5 kg) are diurnal (Hendry et al., 2023; Mohd‐Azlan et al., 2023; Nakabayashi et al., 2021), although one study has suggested they are primarily nocturnal (Grassman Jr. et al., 2005), and another showed crepuscular activity (Hearn et al., 2018). Sunda clouded leopards (*Neofelis diardi*; 11–25 kg) have shown variation among previous studies that describe diel activity, from cathermal (Hearn et al., 2018; Mohd‐Azlan et al., 2023) to crepuscular (Widodo et al., 2022). Sunda leopard cats (*Prionailurus javenensis*; 2–8 kg) have been understudied but are most often considered to have a nocturnal diel pattern (Hearn et al., 2018; Mohd‐Azlan et al., 2023; Nakabayashi et al., 2021).

It is important to investigate the abundance and diel activity patterns of sympatric species, especially when they are closely related taxonomically, as the results can identify which ecological mechanisms are enabling coexistence (Hearn et al., 2018). For example, to avoid the negative effects of competition (e.g., intraguild predation or kleptoparasitism), sympatric carnivores are known to alter their spatial and diel activity patterns, thus allowing them to share a similar prey base but to avoid one another in space and time (Allen et al., 2024; Di Bitetti et al., 2010; Monterroso et al., 2014). Many carnivore species also adjust their activity in response to anthropogenic activity and disturbance (Brodie et al., 2015; Gaynor et al., 2018; Nickel et al., 2020). Despite the ecological importance of the spatial and temporal patterns among these felids, current information is too limited in many areas of the Earth, especially across Borneo (Nakabayashi et al., 2021).

We used cameras placed by the Smithsonian Institution in Sabah, Borneo (see McShea et al., 2024) to assess the detection rates and diel activity patterns of felids detected during the monitoring to better understand their ecology and behavior and build on the previous research on these felids (e.g., Hearn et al., 2018; Hendry et al., 2023; Mohd‐Azlan et al., 2023; Nakabayashi et al., 2021; Sastramidjaja et al., 2015). We first calculated the detection rates of each felid as a relative measure of abundance as a reference for future studies. We then assessed the diel activity of each felid, hypothesizing that each felid would vary in their diel activity based on competition and the diel activity of their prey. Principally, we predicted that the Sunda clouded leopard's smaller competitors, particularly bay and marbled cats, would exhibit different diel activity patterns, potentially to avoid interactions with Sunda clouded leopards (Hearn et al., 2018). Although leopard cats are also smaller than the Sunda clouded leopards, they share little dietary and habitat niche overlap, we therefore predicted that their diel activity would be similar as the leopard cats should not need to adjust their diel activity patterns to avoid interactions (Hearn et al., 2018).

## 2 MATERIALS AND METHODS

### 2.1 Study area

Camera traps were set by the Smithsonian Institute to monitor wildlife across Tawau Hills Park, Kinabalu Park, and Mount Tambuyukon, all in the Malaysian state of Sabah (McShea et al., 2024). Sabah (73,631 km^2^) is the northernmost state of Borneo and is characterized by rugged landscapes that merge into coastal alluvial plains (Hearn et al., 2019). Sabah has a relatively consistent year‐round climate (27–34°C) with high humidity and experiences a high amount of rainfall (1800–4000 mm per year) across two monsoon seasons (November–March and May–September) (Isa & Wong, 2007). Tawau Hills Park and Kinabalu Park are fully protected forest areas (Hearn et al., 2019), although most of the remaining forest was selectively logged at some point before 2010, with dipterocarps initially the main species targeted (Reynolds et al., 2011). Tawau Hills Park (280 km^2^) is mostly lowland dipterocarp rainforest but is surrounded by cacao and oil palm plantations (Huaimei et al., 2019). Within the park, 169 species of flowering plants, 69 mammal species, 240 avifauna species, and 54 species of reptiles have been documented (Abu et al., 2015). Kinabalu Park (754 km^2^) is a center of plant diversity known for having over 5000 species of plants, including the greatest concentration of orchid species on Earth with 90 species endemic to the park alone (van der Ent et al., 2015). Eighty species of mammals have been documented on Mt. Kinabalu within the park (Nor, 2001). Mt. Tambuyukon is also inside Kinabalu National Park and is the third highest peak in Borneo (2579 m elevation), yet is relatively unexplored scientifically (Camacho‐Sanchez et al., 2019). Lowland dipterocarp forest dominates Mt. Tambuyukon up to around 1200 m elevation, switching to low productivity forest at around 1400 m, and cloud forests at around 2000 meters where orchids, climbing bamboos, epiphytes, pitcher plants (Nepenthes sp.), and mosses are abundant (Camacho‐Sanchez et al., 2019). A total of 44 mammal species have been documented on Mt. Tambuyukon (Hawkins et al., 2018), including the endemic summit rat (*Rattus baluensis*) (Camacho‐Sanchez et al., 2018).

### 2.2 Field methods

Camera traps were set at 51 sites by the Smithsonian Institution from 2016 to 2019 for varying amounts of time ($\bar{x}$ = 195.3 days ± SD = 122.0, range 20–785), encompassing all months of the year (Boyce, 2024). Camera traps were placed parallel to the ground at approximately knee height (Boyce, 2024), and along an elevational gradient (McShea et al., 2024). The camera trap images were reviewed for accuracy by a biologist (Kays et al., 2022), and the data used in this manuscript is freely available at http://n2t.net/ark:/63614/w12004603.

### 2.3 Statistical analyses

We used program R (version 4.2.3; R Core Team, 2023) to perform our statistical analyses. we combined consecutive detections of the same species at the same site within 30 min of each other into the same event to reduce pseudoreplication (Avrin et al., 2023; Monterroso et al., 2014). We calculated detection rates (relative abundance) using the number of independent events of each detected felid as:

$$\text{Detection rate}=\left(\text{Events}/\text{Trap nights}\right)\;x\;100.$$

To quantify the activity patterns of felids, we used kernel density estimation methods (Ridout & Linkie, 2009). We transformed the start time of each event to radians corresponding to sun time in the study area, and then used the *overlap* package (Meredith & Ridout, 2017) in program R to fit the data to a circular kernel density to estimate the distribution of activity levels during each time of day.

## 3 RESULTS

Felids were documented 55 times across all cameras and 9958 trap nights. Borneo bay cats were documented at 10 cameras (n_events_ = 12, detection rate = 0.20 ± 0.61), while marbled cats were documented at 5 cameras (n_events_ = 5, detection rate = 0.14 ± 0.71). Sunda clouded leopards were documented at 6 cameras (n_events_ = 15, detection rate = 0.47 ± 1.67), while Sunda leopard cats were documented at 9 cameras (n_events_ = 20, detection rate = 0.25 ± 0.71). Flat‐headed cats were not documented. There was very little spatial overlap among the documented felids, with given pairs of species overlapping at either no camera sites or one camera site with other species (Table 1).

**TABLE 1:** The spatial overlap among the four documented felids at the 51 camera trap sites in Sabah, Borneo.

	Borneo Bay cat	Leopard cat	Marbled cat	Sunda clouded leopard
Borneo Bay Cat	–	1	1	1
Leopard Cat	2.0%	–	0	0
Marbled Cat	2.0%	0.0%	–	1
Sunda Clouded Leopard	2.0%	0.0%	2.0%	–

*Note*: The upper comparisons report the number of cameras where overlap occurred, while the lower numbers report the same information as a percentage.

**FIGURE 1:**
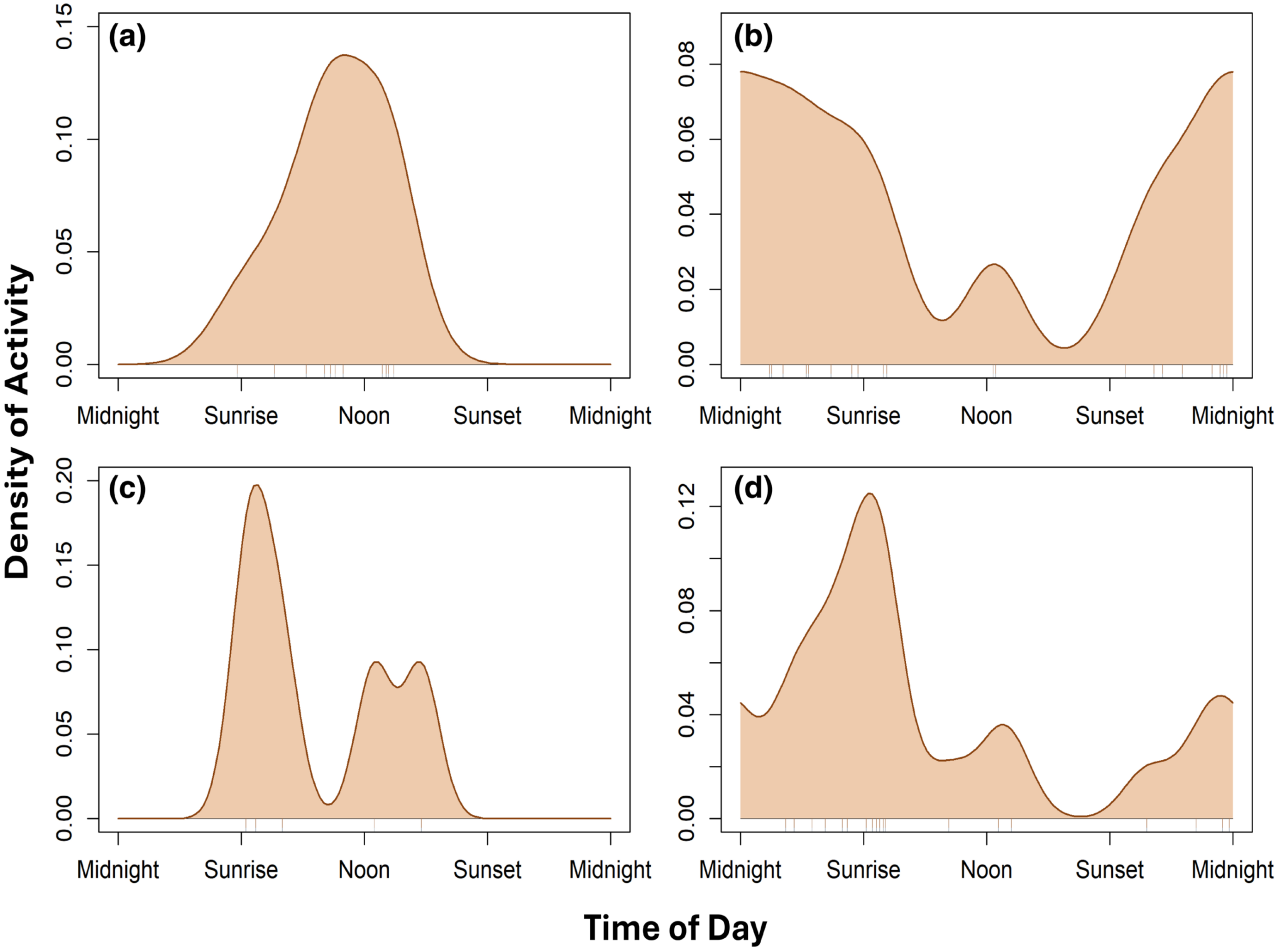
Diel activity adjusted for solar time for the four documented felids in Sabah, Borneo: Borneo bay cat (a), Sunda leopard cat (b), marbled cat (c), and Sunda clouded leopard (d).

Borneo bay cats had a diurnal activity pattern, with a peak slightly before noon (Figure 1). Marbled cats had a cathermal activity pattern, with a peak at sunrise and a smaller peak in the afternoon (Figure 1). Sunda clouded leopards had a cathermal activity pattern, with a peak around sunrise and a smaller peak around midnight (Figure 1). Sunda leopard cats had a nocturnal activity pattern, with a peak around midnight but sustained activity throughout the night (Figure 1).

## 4 DISCUSSION

Our study adds baseline information on detection rates and diel activity to previous research for understudied felid species in an area of conservation concern in Borneo (e.g., Hearn et al., 2018; Hendry et al., 2023; Mohd‐Azlan et al., 2023; Nakabayashi et al., 2021; Sastramidjaja et al., 2015). Borneo is being rapidly developed, with untold effects on wildlife from logging, road development, and conversion of forests to plantations (Cushman et al., 2017; Gaveau et al., 2016; Ocampo‐Peñuela et al., 2020). Studies are therefore needed for developing informed conservation strategies for wildlife, especially carnivores. We detected Sunda leopard cats, Sunda clouded leopard, Borneo bay cats, and marbled cats, but not flat‐headed cats (which are listed as Endangered by the IUCN; Wilting et al., 2015). Aligning with our predictions, Borneo bay cats and marbled cats exhibited different diel activity patterns to Sunda clouded leopard, suggesting temporal displacement to avoid competition and predation; while Sunda leopard cats and Sunda clouded leopards had broadly similar diel activity patterns, suggesting Sunda leopard cats may not use temporal displacement to avoid costly interactions.

The detection rates we observed were generally similar to those reported in other studies. Mohd‐Azlan et al. (2023) reported 681 leopard cat detections and 21 Bornean bay cat detections in Sarawak, yet this was from 845 camera stations over a 17‐year period, indicating a significantly lower detection rate for both species than we report here. However, flat‐headed cats were detected 11 times, which were completely absent from our study. Two studies have reported the presence of felids from camera trap surveys in Sabah (Hearn et al., 2018; Nakabayashi et al., 2021), both reporting all five felid species, but mostly in slightly lower numbers than we report; however, Hearn et al. (2018) reported high leopard cat and moderate Sunda clouded leopard detection rates at specific sites. Collectively, these results suggest that felids are still occupying many areas in the northern parts of Borneo, but that these populations are likely small, fragile, and often isolated from one another. Future conservation strategies should therefore consider state‐ and site‐specific planning to reverse the habitat losses in certain areas and improve connectivity between populations.

Sunda clouded leopards are listed as Vulnerable by the IUCN (Hearn et al., 2016) and are an important species for both their conservation and understanding of their ecological effects. Sunda clouded leopards are only found on the islands of Borneo (where it is the apex predator) and Sumatra (where it is subordinate to tigers). As such, Sunda clouded leopards could be a model species for understanding the effects of ‘de facto’ apex carnivores (e.g., Avrin et al., 2023) because their effects on other species likely vary between islands. Sunda clouded leopards are generally 4.5–6.6 times larger than other felids in Borneo (Hearn et al., 2018), and subordinate species likely adjust their spatial and temporal activity around them. In our study in Sabah, Sunda clouded leopards had a cathermal activity pattern, with a peak around sunrise and a smaller peak around midnight. This may be due to differences among sexes–as males have been documented as being more crepuscular or nocturnal and females more nocturnal, while also exhibiting spatial partitioning as well (Hearn et al., 2018). We did not have information to distinguish easily between sexes, but this temporal partitioning among sexes has also been documented in other felids (Allen et al., In press). Although predators, including Sunda clouded leopards also often adjust their diel activity to overlap with their most abundant prey (Hearn et al., 2018).

Mainland leopard cats (*Prionailurus bengalensis*) are relatively widespread and well‐studied, and are listed as Least Concern by the IUCN (Ghimirey et al., 2023); but Sunda leopard cats (our most frequently detected felid) are severely understudied, including not having an assessment by the IUCN on their conservation status, despite only being found on a limited number of islands. The main reason for the lack of studies is likely that Sunda leopard cats used to be considered the same species as Mainland leopard cats, but there is a lack of information on these felids since they have been split taxonomically into separate species. Our analysis concurred with the nocturnal pattern shown in the only previous study of Sunda leopard cats (Mohd‐Azlan et al., 2023), but would benefit from additional studies. Sunda leopard cats may be the most widespread felid in Borneo (were by far the most frequently detected in surveys by Mohd‐Azlan et al., 2023) and could be a means for large‐scale abundance studies that would provide ‘by‐catch’ information on many other species.

Our findings generally support the previous findings for the smallest felids, Borneo bay cats, and marbled cats. Borneo bay cats are listed as Endangered by the IUCN (Hearn et al., 2015), and are endemic to Borneo, having been rediscovered in 1992 after not being seen for over 60 years (Sunquist et al., 1994). Accordingly, Borneo bay cats are one of the least‐studied felids in the world and worthy of much more attention. We found they had diurnal diel activity, which supports the findings of previous studies (Hearn et al., 2018; Mohd‐Azlan et al., 2023; Sastramidjaja et al., 2015). Marbled cats are listed as Near Threatened by the IUCN (Ross et al., 2016), and had peaks at sunrise and afternoon, which may have been an artifact of our low sample sizes, as most previous studies have suggested they have a diurnal activity pattern (Hendry et al., 2023; Mohd‐Azlan et al., 2023; Nakabayashi et al., 2021). Both species would benefit from greater study to understand their ecology and drivers of abundance, and this may be possible through direct observations or other methods given their diurnal activity.

Camera traps have made the rigorous documentation of cryptic carnivores much easier for wildlife biologists; however, our study highlights that it remains difficult to document these felids in Borneo. In a similar study of a greater extent, Mohd‐Azlan et al. (2023) never documented all five felids in the same study site across the 31 study sites they assessed. Given that it is not surprising that we only documented four felids and failed to detect flat‐headed cats, which are a wetland‐specialist and often less‐detected than other felids (Mohd‐Azlan et al., 2023). Future research may need to focus on specific sites to improve our knowledge of flat‐headed cats, such as along the tributaries of the Kinabatangan and in lowland swamp forests where sightings have been more frequent (Wilting et al., 2010). We also had a general lack of sample sizes, which limited our ability for complex statistical analyses. We hence used detection rates as a proxy for true abundance that has potential limitations, and the management and conservation of felids in the system would benefit from rigorous abundance estimates across time in future studies. However, we again highlight that our numbers are comparable to those reported in Sabah (Hearn et al., 2018; Nakabayashi et al., 2021) and Sarawak (Mohd‐Azlan et al., 2023), suggesting that the populations of these cryptic felids in the northern part of the island are potentially in a fragile state. Nevertheless, our study provides insights that underscore the importance of using rigorous methods to provide information for evidence‐based management and conservation strategies to help preserve biodiversity and ecosystem integrity in Sabah, Borneo.

In order to improve our understanding of the status and ecological needs of these sympatric felid species, camera trapping surveys should consider focusing on locations where we know these species are in greater abundance (e.g., lowland swamp forests for flat‐headed cats). By increasing the density of cameras in these locations, detection rates will be higher, facilitating more complex analyses, such as multispecies occupancy or spatiotemporal overlap among co‐occurring predators and prey. Results from these types of surveys can infer the critical information we need to understand the realized niches of these species. For example, by investigating the patterns of spatial and/or temporal avoidance between species, we should be able to predict the preferred prey of each felid species (Allen et al., 2021), identify the dynamics of exploitative and interference competition between them (Evers et al., 2022), and identity how each species responds to specific anthropogenic disturbances (Nickel et al., 2020). Collectively, this information can then be applied in other locations where population sizes are lower, informing adaptive and targeted management strategies that protect important resources (i.e., prey species) and predator species that have outsized roles in facilitating biodiversity (e.g., providing carrion) and maintaining the health of the ecosystems (LaBarge et al., 2022). It is important to share findings from similar studies with the scientific community, especially for understudied species of conservation concern. In this light, we encourage scientists to make data on rare species publicly available for analysis and support of conservation and management. This is especially important for felids that are likely a good proxy for ecosystem health and are charismatic species that are often used as flagship species for conservation in the area (Mohd‐Azlan et al., 2023). In some cases, basic biology data are missing for many felids (e.g., preferred prey species of Sunda clouded leopards or Borneo bay cats), and it is important to fill these gaps for effective conservation. At the same time, intraguild interactions are likely to affect the habitat use, prey selection, and diel activity of each other (e.g., Hearn et al., 2018), so more complex analyses of the carnivore guild are needed in understudied regions of the world.

## AUTHOR CONTRIBUTIONS

**Maximilian L. Allen:** Conceptualization (equal); formal analysis (lead); methodology (equal); writing – original draft (lead); writing – review and editing (supporting). **Andrew T. L. Allan:** Conceptualization (equal); formal analysis (supporting); methodology (equal); writing – original draft (supporting); writing – review and editing (lead).

## FUNDING INFORMATION

Maximilian Allen received funding for this work from the Illinois Natural History Survey, the Prairie Research Institute, and the University of Illinois. Andrew Allan received funding for this work from The Leverhulme Trust (ECF‐2023‐318).

## CONFLICT OF INTEREST STATEMENT

The authors declare they have no conflicts of interest.

## Data Availability

The data used in the analyses is available from the Smithsonian Institution via Wildlife Insights at: http://n2t.net/ark:/63614/w12004603
